# Novel Instruments for Percutaneous Biportal Endoscopic Spine Surgery for Full Decompression and Dural Management: A Comparative Analysis

**DOI:** 10.3390/brainsci10080516

**Published:** 2020-08-04

**Authors:** Young-Ho Hong, Seung-Kook Kim, Dong-Won Suh, Su-Chan Lee

**Affiliations:** 1 Department of Spine Center, Barun-Sesang Hospital, 5, Yatap-ro 75 Beon-gil, Bundang-gu, Seongnam-si, Gyeonggi-do 13497, Korea; since7999@naver.com (Y.-H.H.); s9187@hanmail.net (D.-W.S.); 2 Himchan UHS Spine and Joint Institute, University Hospital of Sharjah, University Street 1, Sharjah 72772, UAE; 3 Joint and Arthritis Research, Orthopaedic Surgery, Himchan Hospital, 120, Sinmok-ro, Yangcheon-gu, Seoul 07999, Korea; scleeos@empas.com; 4 Department of Pharmaceutical Medicine and Regulatory Sciences, College of Medicine and Pharmacy, Yonsei University, 85, Songdokwahak-ro, Yeonsu-gu, Incheon 21983, Korea

**Keywords:** biportal endoscopic spine surgery, dural protection, endoscopic decompression, percutaneous biportal endoscopic spine surgery, unilateral biportal endoscopy

## Abstract

Background: Post-laminectomy syndrome is a common cause of dissatisfaction after endoscopic interlaminar approach. Our aim was to evaluate the efficacy and safety of our two newly designed instruments for laminotomy, a dural protector attached to the scope and a knot pusher for water-tight suturing of the incidental dural tears. Material and Methods: This was a multicenter evaluation. Efficacy was quantified as the pre-to-postoperative improvement in pain (visual analog scale), disability (Oswestry Disability Index), patient satisfaction (modified MacNab score), and length of hospital stay. Safety was quantified by the incidence and location of dural tears, rate of revision, and radiological outcomes. Outcomes were evaluated between the control (before instrument development) and experimental (after instrument development) groups. Results: There was a significant improvement in leg pain in the experimental group (*p* = 0.03), with greater patient satisfaction in the control group (*p* < 0.01). There was no incidence of dural tears in the area of the traversing and exiting nerve roots in the experimental group. Water-tightness of sutures was confirmed radiologically. Conclusion: The novel dural protector and the knot pusher for water-tight sutures improved the efficacy and safety of decompression and discectomy; however, a prolonged operative time was a drawback.

## 1. Introduction

Post-laminectomy syndrome is a common cause of dissatisfaction after spinal surgery, with remnant or aggravated symptoms reported in 10 to 60% of cases [1,2]. Various factors can contribute to unfavorable outcomes after spinal surgery, including recurrent disc protrusion, persistent foraminal stenosis, incidental nerve injury combined with a dural tear, insufficient decompression, and instability. Minimally invasive spinal surgery, which includes endoscopic surgery, provides various advantages over an open approach, including preservation of the normal tissue, a small operative scar, shorter hospital stays, and less postoperative pain [3,4]. Percutaneous biportal endoscopic spine surgery (PBES), a variant of endoscopic spinal surgery, has been used for the treatment of various types of spinal conditions, ranging from discectomy to interbody fusion, and has been shown to reduce the rate of insufficient decompression [5,6]. However, the lesser amount of muscle dissection and limitation in the type of instruments available have been identified as drawbacks of PBES, with the risk of post-laminectomy syndrome being higher with PBES than with other endoscopic approaches resulting from insufficient decompression [7]. Furthermore, PBES is a single-handed procedure that makes dural protection and achievement of water-tight dural tear repair impossible. Although there exists controversy concerning the most appropriate treatment for dural tears, water-tight suturing is considered appropriate for incidental tearing during surgery to prevent the development of a pseudomeningocele and other complications [8,9]. Recent advances in endoscopic systems, including the scope retractor for dural protection and water-tight suturing systems, could allow full decompression to be achieved under endoscopic guidance. Nonetheless, to the best of our knowledge, these new devices have not been previously evaluated. Therefore, the aim of our study was to describe our newly designed instruments for percutaneous biportal endoscopic spine surgery (PBES), a dural protector attached to the scope and a knot pusher to strengthen the water-tight sutures used to repair incidental dural tear, to retrospectively evaluate the efficacy and safety of using these instruments for full decompression, and to determine whether these novel instruments could improve postoperative outcomes.

## 2. Materials and Methods

### 2.1. Statement of Ethics

All procedures involving human participants were in accordance with the ethical standards of the institutional research committee (169684-01-201912-09) and with the 1964 Helsinki Declaration and its later amendments or comparable ethical standards. Informed consent was obtained from all individual participants included in the study.

### 2.2. Study Design and Population

This was a retrospective, multicenter, cross-sectional study performed between July 2018 and July 2019. The sample size was calculated for a parallel design using G-power for Windows (version 3.1.9.4; Brunsbüttel, Germany). Two endoscopic surgeons, with experience of >200 cases, at two centers (Bareun-sesang Hospital, Kyung-ki, Korea, Himchan Hospital, Incheon, Korea) participated. The inclusion criteria for the patient group were as follows: diagnosis of ipsilateral or bilateral degenerative intracanal stenosis and/or intracanal herniated lumbar disc; persistent intractable symptom, such as severe radiating pain or neurogenic intermittent claudication within 30 min of walking; neurological symptoms and signs for a duration >6 weeks and evidence of a radiological lesion correlating with clinical symptoms. Patients with the following conditions were excluded: tumor or infectious disease; loss to follow-up within 6 months; presence of a spondylolisthesis grade >II; evidence of significant instability on dynamic motion assessment and presence of foraminal or extra-foraminal pathology.

### 2.3. Operative Technique

**Figure 1:**
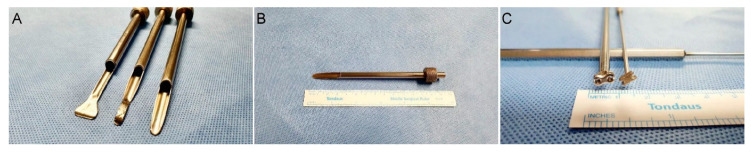
Novel instruments developed for dural protection during biportal endoscopic surgery (**A**,**B**), and a pusher for endoscopic suturing (**C**).

PBES was performed with patients in the prone position on a radiolucent spinal table, after epidural, spinal, or general anesthesia, as appropriate for the patient, provided by an anesthesiologist. Antiseptic skin preparation was completed using betadine and alcohol. For PBES, two 0.7 cm portals were used, one for the scope and one for the instruments. The size of the scope protector, used for dural protection (Figure 1A,B), and the knot pusher, for durotomy repair (Figure 1C), was selected on an individual patient basis. Water pressure was maintained at 100 cm H_2_O (73.55 mmHg, 1 m above the operative table) without use of an infusion pump.

**Figure 2:**
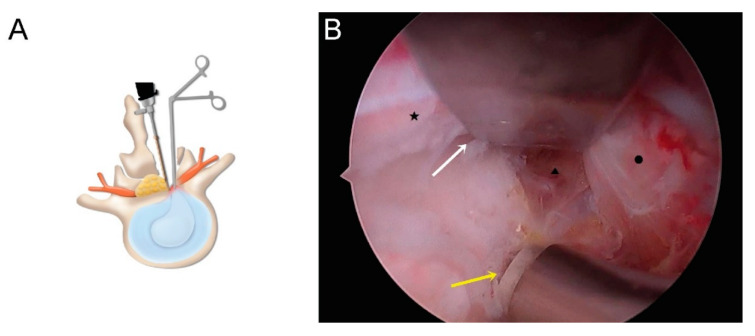
(**A**) Schematic illustration of ipsilateral decompression and discectomy. (**B**) Operative field for discectomy with nerve protection (white arrow: dural protector; yellow arrow: coagulation electrode; ★: thecal sac; ▲: disc; ●: traversing root).

After identifying the inter-laminar space using a muscle detacher, a radiofrequency ablator, and a muscle shaver, a partial hemi-laminotomy was performed using an osteotome and electric drill. For bilateral pathology, we used either an ipsilateral (IPA) or contralateral (CLA) approach, as feasible, for unilateral decompression. For bilateral pathology, both the IPA and CLA approaches were used. In the dural protector group, the traversing root and the thecal sac were protected during ipsilateral lateral recess decompression and discectomy using an IPA approach (Figure 2A,B). 

**Figure 3:**
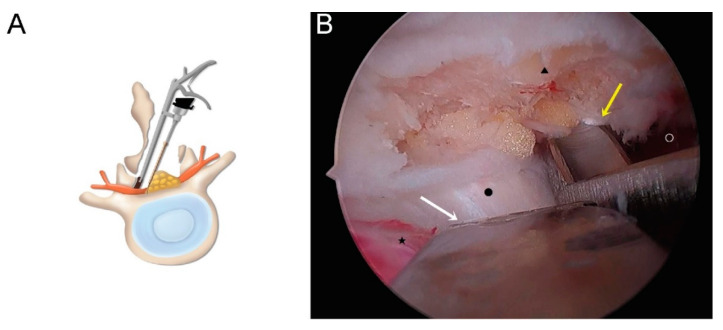
(**A**) Schematic illustration of contralateral decompression. (**B**) Operative field for contralateral facet decompression with nerve protection (white arrow: dural protector; yellow arrow: facetectomy tool; ★: thecal sac; ▲: superior articular process; ●: disc; ○: traversing root).

When using a CLA approach, after sublaminar bony decompression, contralateral facet and lateral recess decompression was performed with protection of the contralateral traversing nerve root and thecal sac (Figure 3A,B).

**Figure 4:**
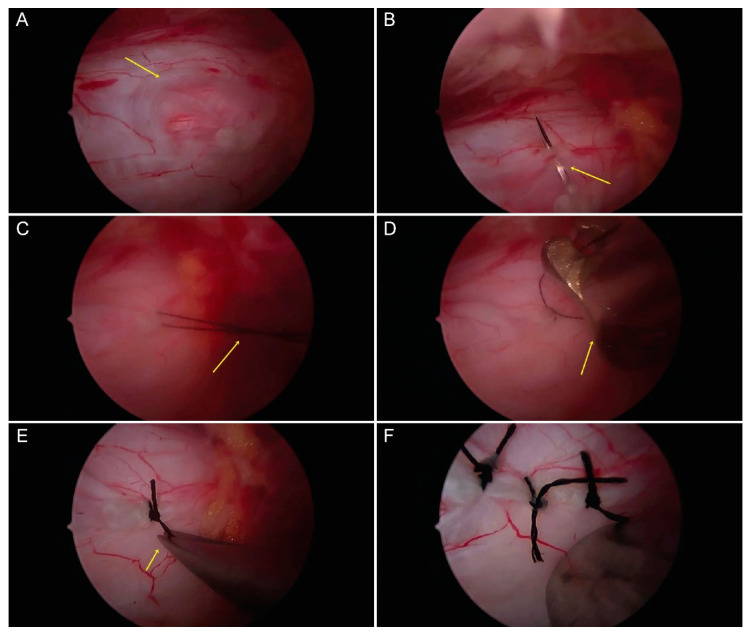
Endoscopic suture using the specially designed pusher. (**A**) An incidental thecal sac lesion during durotomy is shown by the yellow arrow. (**B**) The 4:0 silk needle used to suture both lateral margins, including the needle holder, shown by the yellow arrow. (**C**) The silk suture used is crossed to form a knot (yellow arrow). (**D**) The pusher is used to strengthen the knot to obtain a watertight suture (yellow arrow). (**E**) The ends of the silk suture are cut using micro-scissors (yellow arrow). (**F**) The completed water-tight suturing is shown.

When incidental durotomy occurred, we decreased the irrigation pressure to 50 cm H_2_O (36.78 mmHg) to prevent intradural water overflow. The specially designed knot pusher was used to place water-tight 4:0 silk sutures close to the tear (Figure 4A–F). After all procedures, a 100-cc drainage bag was placed in situ to prevent the development of an epidural hematoma. A one-point skin suture, using 3:0 nylon, was used to close both portal incisions.

### 2.4. Evaluation and Follow-Up

For analysis, outcomes were compared between patients for whom the dural protector, attached to the scope, was used (experimental group) and those for whom the surgery was performed without the dural protector (control group). The following variables were compared between the two groups: type of surgery; preoperative medical condition (quantified using the American Society of Anesthesiologists Physical Status score, ASA-PS, I, healthy, II, mild to moderate, III, severe); grade of stenosis based on magnetic resonance imaging, MRI), Grade 1 (minor), 2 (moderate), 3 (severe), 4 (extreme) [10]; operative time (minutes); length of hospital stay (days); duration of follow-up; change in pre- to postoperative pain score, quantified using a visual analog scale (VAS, 0–10), and disability, quantified using the Oswestry Disability Index (ODI, 0–100%); patient satisfaction, quantified using the MacNab scale (excellent, good, fair, or poor); rate of re-operation (%); incidence rate of dural tears, including the location of dural tears; and the radiological outcomes of treatment.

### 2.5. Statistical Analysis

Between-group comparisons of continuous baseline variables (age, operative time, length of hospital stay, follow-up duration) and VAS and ODI scores were evaluated using unpaired Student’s *t*-test. Categorical variables (sex, rate of revision, and incidence rate of durotomy) were compared between the experimental and control groups using the chi-squared test. Symptom improvement was evaluated using a paired *t*-test. The type of surgery, ASA-PS, MRI grading, modified MacNab score, and distribution of incidental durotomy were compared between the groups using a linear-by-linear association method and chi-squared test. All statistical analyses were performed using R package software (version 3.6.1 for Windows; R Foundation for Statistical Computing, Vienna, Austria). A *p*-value < 0.05 was considered statistically significant.

## 3. Results

### 3.1. Baseline Characteristics

**Figure 5:**
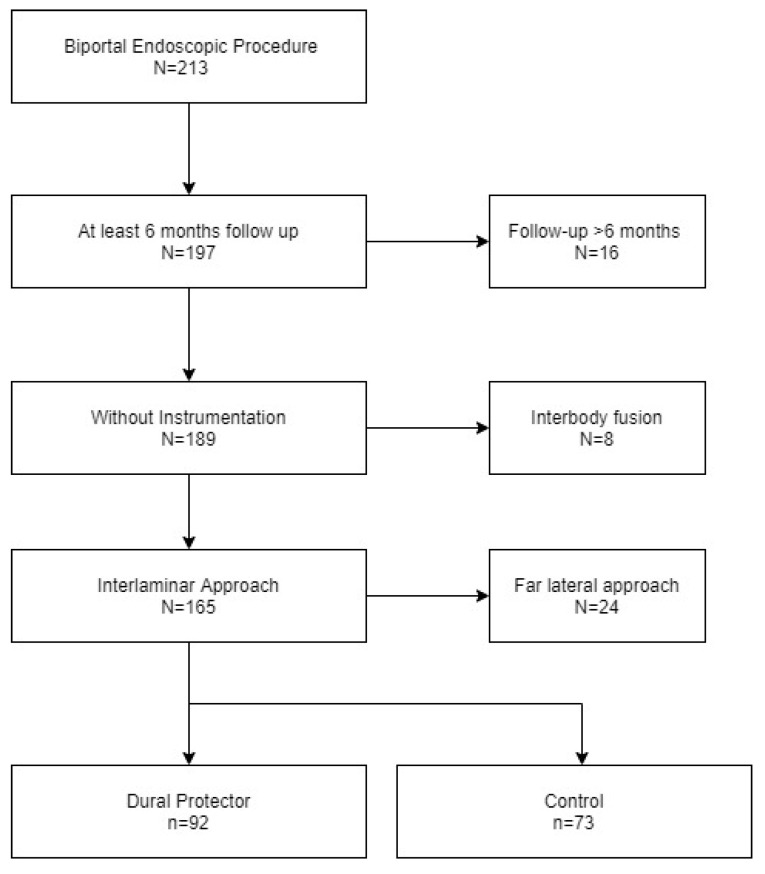
Study population after exclusion.

Over the 12 months of the study period, 213 patients underwent PBES for lumbar decompression. Among these, the following were excluded: 16 lost to follow-up at <6 months; 8 who underwent biportal endoscopic fusion; and 24 who were treated using a far lateral approach. After exclusion of these cases, our analysis was based on the data from 165 patients treated for an intracanal pathology by PBES. (Figure 5) Among these 165 cases, 92 underwent PBES with a dural protector (experimental group) and 73 without (control group). Demographic and baseline data of the two groups are summarized in Table 1 No significant difference in the distribution of age, sex, preoperative medical conditions, and MRI grading was observed between the two groups. Discectomy was more frequently performed in the experimental group (29.70%) than in the control group (1.21%) (*p* < 0.01). Operative time was significantly longer in the experimental group (81.52 ± 48.75 min) than in the control group (60.41 ± 25.51 min) (*p* < 0.01).

### 3.2. Efficacy and Safety of the Novel Instruments

**Figure 6:**
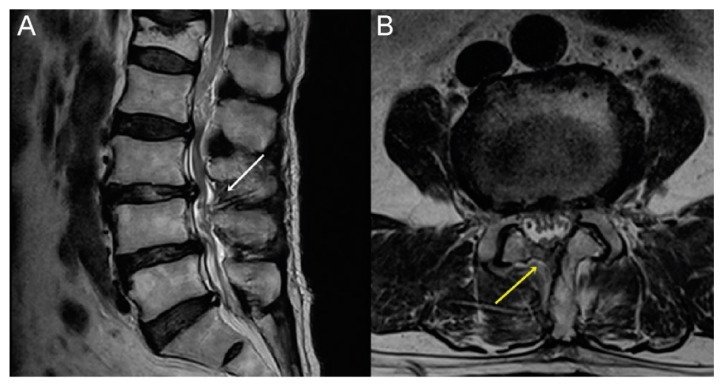
Radiological outcome after endoscopic suturing. (**A**) T2-weighted sagittal (white arrow: suture site) and (**B**) T2-weighted axial magnetic resonance images (yellow arrow suture site). There is no evidence of residual cerebrospinal fluid.

Outcomes of the procedure are presented in Table 2. Patient satisfaction was higher in the experimental group (excellent 21.82% and good 33.94%) than in the control group (excellent 1.21% and good 43.03%) (*p* < 0.01). With respect to efficacy, the experimental group exhibited significant improvement in leg pain, with a decrease in the VAS score (from 5.64 ± 1.53 in the experimental group to 5.06 ± 1.75 in the control group) (*p =* 0.03). Nevertheless, there was no difference in the ODI score between the experimental and control groups (18.00 ± 7.72 and 16.01 ± 5.88, respectively; *p =* 0.07), rate of re-operation (4.34% and 4.11%, respectively; *p =* 0.62), and rate of incidental durotomy (4.35% and 10.96%, respectively; *p =* 0.09). Although the location of dural tears was not different between the two groups (*p =* 0.22), there was no incidence of tears in the area of the traversing and exiting nerve roots in the experimental group. As for adverse outcomes in the experimental group, two patients required endoscopic suturing (one was treated with rest and the other required a fibrin sealant patch), neither of whom presented with cerebrospinal leakage (Figure 6A,B). One case was a dural tear sustained during ipsilateral laminotomy performed using an osteotome, treated with 3-point suturing, requiring an additional 15 min of operative time. The other case was a tear in the area of the thecal sac sustained during contralateral decompression using a Kerrison punch, treated, again, with 3-point suturing, requiring an additional 17 min of operative time. Both patients recovered with a 2-day period of bedrest, without additional cerebrospinal fluid (CSF) leakage. In the control group, eight patients required further treatment (four treated with bedrest and four requiring a fibrin sealant patch). Furthermore, cases in the control group recovered without CSF fluid leakage or pseudomeningocele formation.

## 4. Discussion

### 4.1. Overall Outcomes

Our newly developed instruments for PBES were safe to perform discectomy and lateral recess decompression. With regard to efficacy, although the operative time was prolonged with the use of our novel instruments, this did not negatively affect the improvement in ODI score after surgery. Moreover, the improvement in pain and patient satisfaction was higher for the experimental than the control group. For repair of incidental durotomy, the use of our new knot pusher provided a safe and water-tight suturing management.

### 4.2. Endoscopic Surgery in Degenerative Spine Disease

Previous studies have reported on the superior efficacy of an endoscopic approach, compared to a microscopic approach, in improving leg pain and patient satisfaction for the treatment of disc herniation [11,12]. Any superior effect of an endoscopic approach for the treatment of spinal stenosis or spondylolisthesis, however, remains an issue of controversy, with a prospective randomized control trial reporting a benefit of a microscopic approach on short-term back pain, measured within one week after surgery, but without a clear benefit of a microscopic approach over the longer term [13]. With regard to fusion surgery as treatment for segmental spinal instability and spondylolisthesis, a retrospective case series [14] and a comparative study [15] indicated that an endoscopic approach was not inferior to a microscopic approach for interbody fusion. Overall, while there exists evidence for the short duration of discectomy, the efficacy of decompression and fusion surgery requires more evidence from randomized controlled comparative studies.

### 4.3. Trials for Overcoming Limitation of Instruments

Overall, limitations in the availability of instruments, the narrow space for bone and ligament resection, and lack of materials to control bleeding and for vertebral fusion have been identified as limitations of endoscopic approaches for spinal decompression and fusion surgery. Various automated drills, osteotomes, and curved Kerrison punches for bone and ligament removal have been introduced [16], providing high-definition image guidance and sufficient decompression. Nevertheless, dural protection has remained a concern. In our study, we report on the new scope protector that we developed to protect neural structures during discectomy or facetectomy. As a distinct advantage, the protector is easily controlled by the surgeon and, thus, protects against excessive traction or mistake by the surgical assistant.

With regard to complications, incidental durotomy during endoscopic surgery has been treated using a fibrin sealant patch, a non-penetrating clip, or conversion to a microscopic approach [9,17]. However, treatment of an incidental durotomy using water-tight Prolene or silk sutures has been shown to provide superior outcomes compared to a fibrin sealant or hydrogel [8]. Meticulous suturing can be expected to yield favorable outcomes and to decrease the rate of conversion to a microscopic approach. This is important as conversion to microscopic surgery requires sacrifice of extensive muscle tissue and extends the operative time.

### 4.4. Other Technical Solutions for a Biportal Technique

Minimally invasive dissection helps with rapid postoperative recovery but does reduce the area of decompressive laminotomy [18]. Customized dissection for additional muscle and ligament detachment can be useful for sufficient decompression. To control bleeding, a small tip radiofrequency coagulator, as well as bone wax of various size, can be used. Fibrin sealant and gel foam can also be applied, if needed. Furthermore, various types of impactors are available for the application of bone chips (or harder materials) in endoscopic fusion surgery [5].

### 4.5. Pros and Cons of Biportal Instruments Compared to Other Endoscopic or Microscopic Techniques

Theoretically, use of a single port technique, with dilation and no detachment of muscles, is less invasive than the PBES procedure [12]. PBES requires two portals, one for the scope and one for the instrument, which overcomes the technical difficulty of using a rigid working canula in the single portal technique. Moreover, a single portal technique requires additional instruments, which increases the cost of the procedure compared to the PBES technique. Furthermore, once introduced, the scope protector can be used for the root retraction procedure without the need for additional soft tissue removal or the need for assistance, the latter reducing the risk of over traction and problems of cooperation between the surgeon and the assistant [19]. In fact, the efficacy and safety of PBES are largely related to the fact that a surgeon can use both hands, with bilateral manipulation of instruments, allowing for adequate nerve protection against over-traction. The flexible working channel used in PBES further offers the possibility of widening the channel, as indicated, depending on the needs of the surgery. Because of this flexibility, various instruments can be developed, without the limitation of space needing to be considered. In this way, the major limitations of an endoscopic approach for spinal surgery can be overcome.

In our study, sufficient decompression laminectomy was achieved without root injury. Bleeding control and manipulation near the thecal sac and nerve root are essential as nerve damage can lead to negative sequelae, poor outcomes and low patient satisfaction [20]. A dural protector is effective in providing better visualization [21] but nerve root protection is needed to support full decompression and safe discectomy. We suspect this is the reason for our increased rate of discectomy and efficacy in all cases of decompression and discectomy. Another study reported on the advantage of PBES to accommodate a scope of varying angle, from 0° to 30°, compared to other endoscopic techniques [22]. In microscopic surgery, the free use of both hands for instrument handling and the sufficient area for dissection are specific strengths of the technique for tumor removal, suturing of incidental dural tears, and for achieving sufficient decompression of severe stenosis, without an additional learning curve for surgeons [23,24]. However, adjustment of the microscope and tilting of the table are needed to access the contralateral area [25]. This requires time and the angle provided by the scope is typically insufficient for appropriate monitoring for contralateral facet lesions. With the PBES technique, both minimally invasive muscle detachment and dilation are possible, which allow for sufficient decompression to be achieved while preserving muscle structure. Moreover, small movements of the endoscope provide a wide range of vision and decompression. Our current study further shows the benefit of PBES in offering nerve protection using the scope protector, rather than relying only on direct vision to achieve this in microscopic surgery.

### 4.6. Effects of the New Instruments on Operative Time

The use of our novel instruments for PBES did prolong the operative time compared to that in the control group. In the absence of using a scope protector, the use of a retractor is avoided to reduce the risk of nerve injury and, hence, decompression is performed without discectomy. However, disc protrusion can result in nerve root compression; thus, the performance of both discectomy and flavectomy can improve surgical outcomes. Moreover, the thecal sac is often injured during contralateral superior facetectomy. The use of the scope protector allowed us to confidently use a retractor for the removal of the hard and calcified disc without the risk of injuring the nerve root. This likely explains the favorable outcomes of the experimental group over the control group. We also need to balance the prolongation in operative time when using our novel tool with the improvement in achieving sufficient decompression when using our tools, as well as the benefit of the protector attachment in allowing more frequent disc removal. These outcomes, which are necessary for a detailed procedure, could be further enhanced with practice and further design development of the instruments.

### 4.7. Limitations of the Study and Future Directions

Owing to the retrospective design of our study, with absence of randomization, bias on the measured efficacy and safety of the PBES procedure using our novel instruments cannot be denied. Although randomization of the surgical approach is not feasible, blinded randomization of measured outcomes would increase the confidence in reported outcomes. In addition, considering the retrospective design, our outcomes may reflect a learning curve. A prospective, large scale, study with a longer follow-up period, would provide the level of evidence needed to inform clinical decisions on the use of our novel instruments for PBES. Furthermore, based on different approaches and technique, a comparison of ipsilateral and contralateral decompression would be valuable. Additional patient-centered assessments could also be helpful to improve the relevance of measured outcomes. Nonetheless, this is the first report on the use of our multifunctional endoscopic and dural suturing system, with evidence of the efficacy and safety of our newly developed instruments for discectomy and lateral recess decompression.

## 5. Conclusions

For PBES, the use of a dural protector, attached to the scope, was efficacious and safe for spinal decompression and discectomy surgery. Furthermore, we were able to achieve water-tight sutures without conversion to a microscopic approach. However, the prolonged operation time when using our instruments was drawbacks. Continued development of various instruments is needed for better outcomes and to continue to decrease the time needed for this procedure.

## Figures and Tables

**Table 1:** Baseline demographics of patients in full endoscopic decompression with or without dural protector groups.

Factors	Total (*n* = 165)	Dural Protector (*n* = 92)	Control (*n* = 73)	*p*-Value
Age, years; mean (SD)	60.59 (13.12)	61.13 (14.19)	59.83 (11.68)	0.50 †
Sex, *n* (%)				0.08 ‡
Male	101 (61.21)	82 (89.13)	19 (26.03)	
Female	64 (38.79)	10 (10.87)	54 (73.97)	
Type of surgery, *n* (%)				0.01 ¶*
Ipsilateral laminotomy	25 (15.15)	11 (6.67)	14 (8.48)	
Contralateral laminotomy	1 (0.61)	0 (0)	1 (0.6)	
bilateral laminotomy, unilateral approach	88 (53.33)	32 (19.36)	56 (33.94)	
Discectomy	51 (31.52)	49 (29.70)	2 (1.21)	
ASA-PS, grade; *n* (%)				0.71 ¶
I (Healthy)	110 (54.55)	45 (27.27)	45 (27.27)	
II (Mild to moderate)	74 (44.85)	47 (28.48)	27 (16.36)	
III (Severe)	1 (0.61)	0 (0)	1 (0.6)	
MRI grading				0.34 ¶
A (Minor)	50 (30.3)	28 (16.97)	22 (13.33)	
B (Moderate)	58 (35.15)	31 (18.79)	27 (16.36)	
C (Severe)	48 (29.09)	27 (16.36)	21 (12.73)	
D (Extreme)	9 (5.45)	6 (3.64)	3 (1.82)	
Number of levels, *n* (%)				0.08 ¶
1	138 (83.64)	74 (44.85)	64 (38.78)	
2	26 (15.76)	18 (10.90)	8 (4.85)	
3	1 (0.61)	0 (0)	1 (0.6)	
Operative time, min; mean (SD)	72.21 (41.40)	81.52 (48.75)	60.47 (25.51)	>0.01 †*
Length of hospital stay, days; mean (SD)	5.82 (3.03)	5.80 (2.53)	6.48 (2.42)	0.08 †
Duration of follow-up, months; mean (SD)	8.52 (3.01)	9.64 (3.36)	7.63 (2.38)	0.11 †

† Student’s *t*-test, ‡ chi-squared test, ¶ Linear-by-linear association; * is statitically significant. (*p* < 0.05). *SD* standard deviation, *ASA-PS* American Society of Anesthesiologists Physical Status, *n* number, *MRI* magnetic resonance imaging.

**Table 2:** Efficacy and safety of percutaneous biportal endoscopic surgery using a protector and pusher.

	Total (*n* = 165)	Dural protector (*n* = 92)	Control (*n* = 73)	*p*-Value
Pain score, mean (SD)				
Pre-VAS	7.09 (1.27)	7.23 (1.05)	6.91 (1.49)	0.12 †
Post-VAS	1.70 (1.05)	1.59 (1.17)	1.84 (0.84)	0.11 †
VAS improvement	5.38 (1.65)	5.64 (1.53)	5.06 (1.75)	0.03 †*
*p*-value	>0.01 ‡*	>0.01 ‡*	>0.01 ‡*	
Disability score, mean (SD)				
Pre-ODI	29.22 (8.10)	31.52 (1.05)	26.33 (6.54)	0.01 †*
Post-ODI	12.10 (4.90)	13.53 (5.20)	10.31 (3.82)	0.01 †*
ODI improvement	17.11 (7.02)	18.00 (7.72)	16.01 (5.88)	0.07
*p*-value	>0.01 ‡*	>0.01 ‡*	>0.01 ‡*	
Satisfaction, *n* (%)				>0.01 ¶*
Poor	2 (1.12)	0 (0)	2 (1.21)	
Fair	3 (1.2)	1 (0.6)	2 (1.21)	
Good	127 (73.94)	56 (33.94)	71 (43.03)	
Excellent	38 (23.03)	36 (21.82)	2 (1.21)	
Rate of revision, *n* (%)	7 (4.24)	3 (4.34)	4 (4.11)	0.62 ¥
Rate of durotomy, *n* (%)	12 (7.27)	4 (4.35)	8 (10.96)	0.09 ¥
Distribution of durotomy, *n* (%)				0.22 ¶
Exiting zone	1 (0.6)	0 (0)	1 (1.21)	
Thecal zone	8 (4.85)	4 (2.42)	4 (2.42)	
Traversing zone	3 (1.82)	0 (0)	3 (1.82)	
Durotomy management, *n* (%)				NA
Rest	5 (3.03)	1 (0.6)	4 (2.42)	
Fibrin sealant	5 (3.03)	1 (0.6)	4 (2.42)	
Endoscopic suture	2 (1.21)	2 (1.21)	0 (0)	

† Student’s *t*-test, ‡ paired *t*-test, ¶ Linear by linear association, ¥ chi-squared test, * is statitically significant. (*p* < 0.05), *SD* standard deviation, *n* number, *NA* not available, *VAS* visual analog scale.
